# Update in collecting duct carcinoma: Current aspects of the clinical and molecular characterization of an orphan disease

**DOI:** 10.3389/fonc.2022.970199

**Published:** 2022-10-04

**Authors:** Cristina Suarez, David Marmolejo, Augusto Valdivia, Rafael Morales-Barrera, Macarena Gonzalez, Joaquin Mateo, Maria Eugenia Semidey, David Lorente, Enrique Trilla, Joan Carles

**Affiliations:** ^1^ Medical Oncology, Vall d´Hebron Institute of Oncology (VHIO), Hospital Universitari Vall d´Hebron, Vall d´Hebron Barcelona Hospital Campus, Barcelona, Spain; ^2^ Pathology Department, Vall d’Hebron University Hospital, Vall d´Hebron Barcelona Hospital Campus, Barcelona, Spain; ^3^ Urology Department, Vall d’Hebron University Hospital, Vall d´Hebron Barcelona Hospital Campus, Barcelona, Spain

**Keywords:** collecting duct (cdRCC), non-clear cell (ncRCC), renal carcinoma (RCC), Bellini carcinoma, clear-cell carcinoma (ccRCC)

## Abstract

Collecting duct renal cell carcinoma (cdRCC), which until recently was thought to arise from the collecting ducts of Bellini in the renal medulla, is a rare and aggressive type of non-clear renal cell carcinoma (ncRCC), accounting for 1% of all renal tumors and with nearly 50% of patients being diagnosed with Stage IV disease. The median overall survival in this setting is less than 12 months. Several regimens of chemotherapies had been used based on morphologic and cytogenetic similarities with urothelial cell carcinoma described previously, although the prognosis still remains poor. The use of targeted therapies also did not result in favorable outcomes. Recent works using NGS have highlighted genomic alterations in *SETD2, CDKN2A, SMARCB1*, and *NF2*. Moreover, transcriptomic studies have confirmed the differences between urothelial carcinoma and cdRCC, the possible true origin of this disease in the distal convoluted tubule (DCT), differentiating from other RCC (e.g., clear cell and papillary) that derive from the proximal convoluted tubule (PCT), and enrichment in immune cells that may harbor insights in novel treatment strategies with immunotherapy and target agents. In this review, we update the current aspects of the clinical, molecular characterization, and new targeted therapeutic options for Collecting duct carcinoma and highlight the future perspectives of treatment in this setting.

## Introduction

Collecting duct renal cell carcinoma (cdRCC), which until recently was thought to arise from the collecting duct in the renal medulla (Bellini duct), is a rare and aggressive type of non-clear renal cell carcinoma (ncRCC). It accounts for approximately 1% of all renal tumors with decreasing incidence (1). The median age at presentation is 59 years, with a male to female ratio of 2:1. Clinical staging at diagnosis varies widely, being 18.9%, 4.2%, 24.1%, and 50.3% for diagnosis at stages I, II, III, and IV, and the OS rates at 1, 2, and 5 years were 55.5%, 39.4%, and 26.8%, respectively; indicating that over 50% of the cdRCC patients die within two years. Distant metastases at disease debut are found in 42% of patients, reflecting the difficulties in the early diagnosis of this sickness. As expected of this aggressive tumor, overall survival in patients with metastatic disease is dismal compared to non-metastatic setting (7 months vs. 53 months), as also compared to ccRCC and other ncRCC subtypes with a significantly higher cancer specific mortality (HR 1.6, p<0.01) (1–3).

Compared to ccRCC, cdRCC seems to be more frequently found in African-American population. However, to date, no other specific risk factors of this entity have been identified (4). There have been significant advances in this entity’s molecular landscape and therapeutic arsenal.

This review aims to show said improvements, emphasizing advances in the molecular landscape of cdRCC and how acquiring this knowledge achieved progress in finding new therapeutic options with the potential to improve survival outcomes.

## Clinical diagnosis and radiology

According to the literature, the clinical presentation of this rare variety of ncRCC does not differ from the other subtypes of renal cell carcinomas (RCC). The most common symptoms are gross hematuria, abdominal pain, weight loss, flank mass, and fatigue. These symptoms are present in more than half of patients at diagnosis. Furthermore, more than 30% of patients debut with metastatic disease, confirming its aggressive biological behavior and having the worst prognosis among all renal tumors (5–8).

Previous cohorts of cdRCC in the SEER database revealed that the 3-year relative survival rates for localized, regional, and distant disease were 93%, 45%, and 6%, respectively. Moreover, information revealed higher stage, higher grade, and poorer prognosis in comparison to clear cell RCC (4, 9). A recent study of 286 cases reported from 2004 to 2018 in the SEER database showed that the median overall survival (OS) for cdRCC patients was 16 months. The proportions of regional lymph nodes and distant metastasis were 40.6% and 42.0%, respectively (1). These results are consistent with the largest report on cdRCC by Sui W et al. (10) where the median survival and the proportion of metastatic patients for the cdRCC cohort were 13,2 months and 70,7%, compared to 122,5 months and 30% of patients for the ccRCC cohort, respectively.

Most cdRCC originate in the renal medulla and infiltrate the cortex and renal pelvis. Unfortunately, no specific radiologic finding helps distinguish cdRCC from other tumors (11). Patients usually undergo an abdominal computed tomography (CT scan), typical findings include medullary location, weak and heterogeneous enhancement, involvement of the renal sinus, infiltrative growth, preserving of renal contour, and cystic component (12). The stroma of cdRCC is highly fibrous and collagenized, giving it a water-rich density. Due to this, the density of cdRCC is higher than the surrounding normal tissues in non-enhanced CT scans. This characteristic differs from renal tumors arising in the renal cortex. However, after administering iodine-contrast, this trait is barely identified compared to surrounding normal renal parenchyma (11).

Pickhardt et al. (13) reported four cases of cdRCC studied with magnetic resonance imaging (MRI). Tumor parenchymal components showed equal signals on T1W1, but lower than those of normal renal parenchyma on T2W1. Similarly, Kato et al. (14) described that the tumor parenchyma showed an isointense or hypointense signal on T2WI, which was thought to be due to hemosiderin deposition. In contrast to larger ccRCC, which tend to have a heterogeneous hyperintense signal on T2WI.

There is scanty information about positron emission tomography (PET) performance. Moreover, most RCCs have low Fluorodeoxyglucose (F-FDG) metabolism, similar to the normal parenchyma in addition F-FDG is excreted *via* the kidneys. However, cdRCC is characterized by high invasiveness and a high rate of metastasis at diagnosis. Therefore, PET/CT could be useful for diagnosing advanced stages and assess the extent of the disease. In this regard, Hu et al. reported in two of six patients a high metabolism in lymph nodes, lungs, pleura, and multiple bone lesions, as frequent dissemination sites. Notably, they also reported a case with multifocal CDC in the same kidney, a quality that may occasionally be observed in these patients (15).

To date, the imaging features of cdRCC are not well characterized.

## Pathology and histology

In 1976, Mancilla-Jimenez et al. described three patients with papillary RCC and atypical hyperplastic changes near the collecting duct epithelium. Later, in 1986, Fleming and Lewi proposed the characteristics of cdRCC and presented six cases with said features. At this moment, the designation of Bellini tract tumors was carried out because the origins of these tumors appeared to arise from the collecting duct epithelium. By 1997, the Heidelberg classification of renal tumors identified five histologic types of renal cancer that included collecting duct carcinoma (16–18).

Histologically, collecting duct carcinomas show, in general, high grade tubular morphology with characteristically infiltrative growth accompanied by stromal desmoplasia. It shows nuclear enlargement with prominent nucleoli. They are predominantly centered in the renal medulla. For its diagnosis it is mandatory to exclude other types of Renal Cell Carcinomas (RCC), such as MiTT translocated RCC (positive TFE3 or TFEB immunohistochemistry-IHC, break apart *in situ* hybridization, FISH), Fumarate hidratase deficient RCC (negative fumarate hidratase IHC), Clear Cell RCC (positive CAIX-Vimentin IHC), renal medullary carcinoma (SMARC-B1 deficiency by IHC) and urothelial carcinomas with glandular differentiation (positive CK7, GATA3, Uroplakin, negative PAX8). Possible metastatic carcinomas must be ruled out as well, such as lung primary carcinomas (TTF1 and napsin positives by IHC) (19).

**Figure 1:**
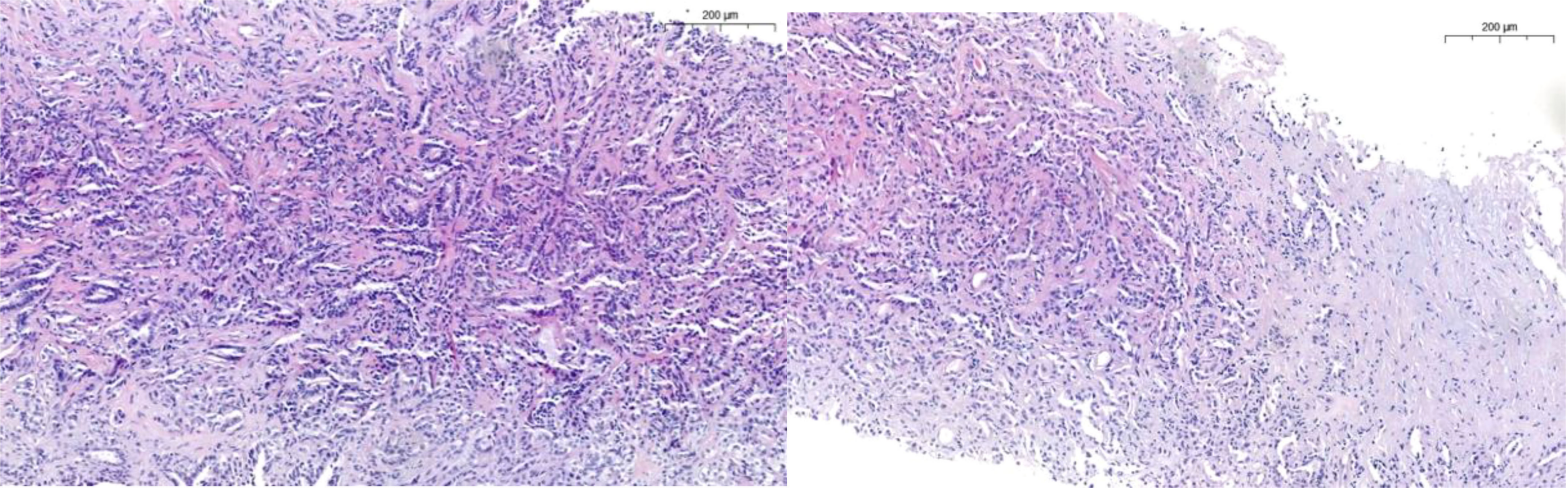
Characteristically tubular pattern in CDC with high grade features such as nuclei enlargement, nucleoli, mitosis, desmoplasia and infiltrative growth.

The International Society of Urological Pathology (ISUP) in 2013 and later the WHO classification in 2016 defined the histologic characteristics of cdRCC, agreeing that it was high-grade by definition and therefore should not be assigned a grade. The main features are outlined in **Table 1** and **Figure 1** (20, 21).

**Table 1:** Pathologic characteristics of Collecting duct Renal Cell Carcinoma.

At least a portion of the tumor involves the medullary region
Predominant formation of tubules
Desmoplastic stromal reaction
Cytological high-grade features
Infiltrative growth patterns
No other renal cell carcinoma subtypes or urothelial carcinoma

## Molecular landscape of cdRCC

The proximity of the collecting duct to the upper urothelial tract led researchers to believe that cdRCC had biological similarities with upper urothelial tract carcinoma (UTUC). Orsola et al. (22) were the first to evaluate this relationship, assessing the clinical, radiologic, and pathologic presentation of three patients with tumors located near the renal papilla. The authors concluded that cdRCC might be distinct from conventional renal cell carcinoma and could share biologic features with UTUC, with therapeutic consequences.

Later studies challenged this assumption. Becker et al. (23) carried out comparative genomic hybridization (CGH) in twenty-nine cdRCC and twenty-six UTUC tumor samples to clarify if genomic alterations between UTUC and cdRCC were similar. cdRCC had a slightly lower number of chromosomal aberrations than UTUC: 4.9/case vs. 5.4/case. In cdRCC samples, the authors found losses in chromosome regions 8p, 16p, 1p, and 9p. Gains were observed in 13q. No amplifications were observed. In contrast, UTUC samples showed amplification of chromosomal regions on 1p, 3p, 6p, and 12q. Fifty percent of samples had losses on 9q. Furthermore, recurrent losses were detected on chromosomes 11, 13, 9p, 17, 10, 3, 8p and 16. Gains were also observed in regions 8q, 7, 1, 5, and 6. The authors concluded that cdRCC had a different genetic pattern than UTUC and may even represent a unique entity among kidney tumors.

**Figure 2:**
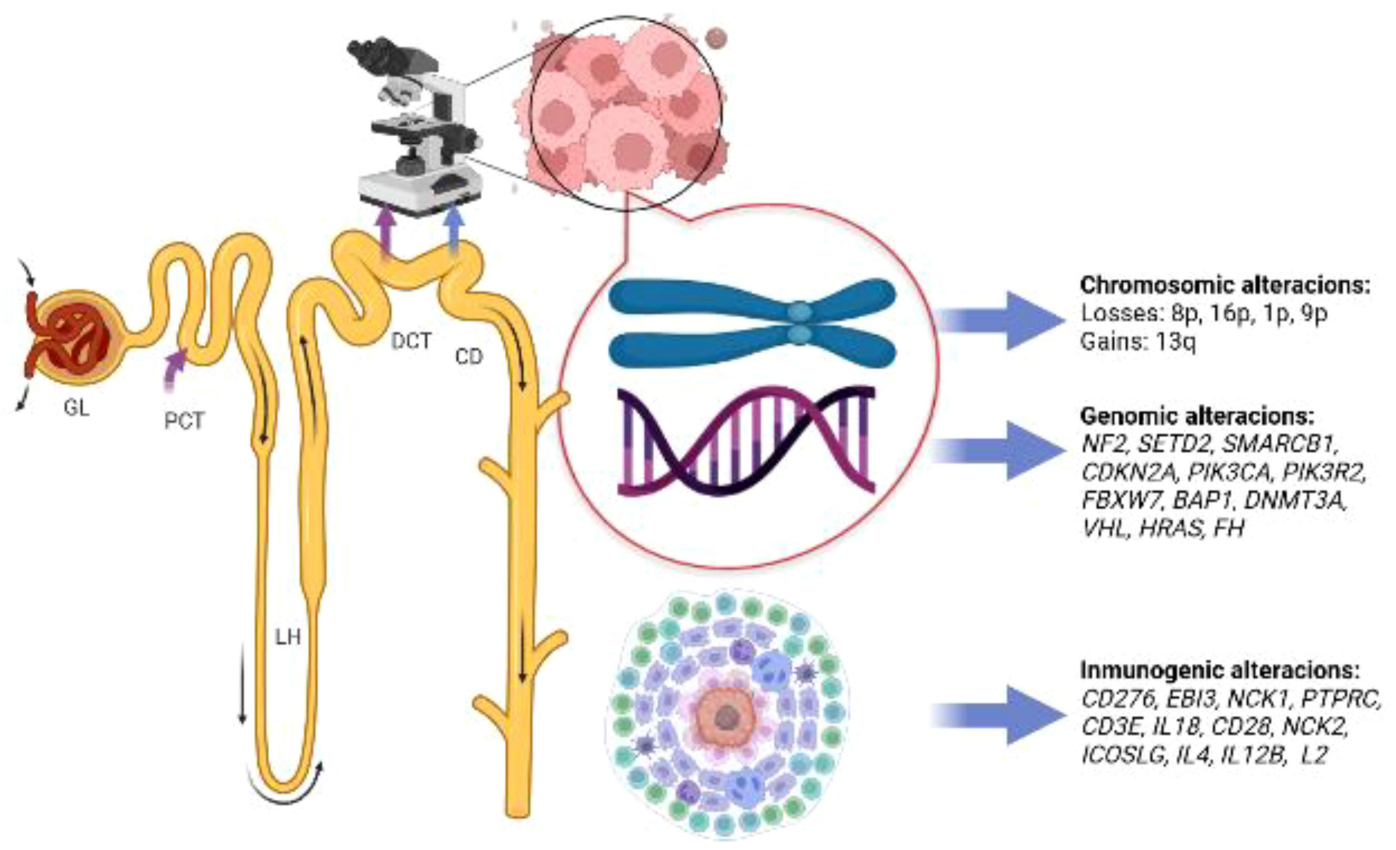
Summary of molecular alterations found in patients with collecting duct carcinoma, glomerulus (GL); proximal convoluted tubule (PCT); loop of Henle (LH); distal convoluted tubule (DCT); collecting duct (CD). Image created with biorender.com.

Pal et al. (24) carried out comprehensive genomic profiling (CGP) with next-generation sequencing (NGS) on seventeen samples of stage III and IV cdRCC patients. Fourteen came from the primary tumor and three from metastatic sites (two lymph nodes and one adrenal gland). Thirty-six genomic alterations were found, with a rate of 2.1 genomic alterations per case. NF2 (29%) and SETD2 (24%) were the most common genomic alterations. The authors also found FH homozygous losses in 22% of patients, observing that FH and SMARCB1 alterations were mutually exclusive to NF2. Notably, some of these genomic alterations were deemed to be clinically relevant, and due to the high number of NF2 mutations found, the authors concluded that mTOR inhibition could be an attractive therapeutic strategy in patients with cdRCC and NF2 mutations. Other possible therapeutic strategies included: histone deacetylase inhibition, EZH2 inhibition, antiangiogenic therapies, and CDK 4/6 inhibition. Other genomic alterations are depicted in **Figure 2**.

To clarify the differences between cdRCC and UTUC and to explore the transcriptome of cdRCC, Malouf et al. (25) performed RNA-sequencing on tumor samples of seventeen cdRCC patients, nine UTUC, and three healthy kidney samples. Unsupervised hierarchical clustering was carried out between the three, and the authors identified two clusters that clearly differentiate cdRCC from UTUC. Furthermore, healthy kidney samples clustered closer to cdRCC than UTUC, highlighting the similarity of cdRCC’s transcriptome to that of normal kidney compared to the urothelium. The authors underwent further studies to determine the nephron site of origin of this entity. Eight distinct regions of the ordinary nephron were compared with the gene expression dataset from cdRCC samples. Findings were consistent with cdRCC originating from the distal convoluted tubule (DCT), differentiating cdRCC from other RCC (e.g., clear cell and papillary) that derive from the proximal convoluted tubule (PCT) (25).

Gene ontology analysis using Database for Annotation, Visualization and Integrated Discovery (DAVID), revealed highly enriched genes related to immune response compared to the normal kidney. Later, Gene Set Enrichment Analysis (GSEA) was used to clarify the deregulated immune pathways. The authors found alterations in the early activation of T lymphocytes, regulation of lymphocyte activation, and T lymphocyte proliferation. Genes expressed in the T cell proliferation pathway included: CD276, EBI3, NCK1, PTPRC, CD3E, IL18, CD28, NCK2, ICOSLG, IL4, IL12B, and IL21. Immune infiltration by CD3+ and CD8+ TILs were found in the tissue slides, especially in metastatic disease. Overall median TIL percentage in cdRCC was 22% for CD3+ TILs and 11% for CD8+. The authors concluded that cdRCC showed a transcriptomic profile closer to renal tumors, has a high level of immune lymphocyte infiltration, and may originate in the DCT (25). Based on these data, immunotherapy appears to be an appealing strategy for these patients.

Regarding differences with other ncRCC, Bratslavsky et al. (26) performed a CGP study to compare the molecular features of cdRCC with two separate cohorts of renal medullary carcinoma (RMC) and metastatic ccRCC. A DNA-sequencing on tumor samples of 46 cdRCC patients, 24 of RMC and 626 ccRCC were performed. The authors showed a significant difference in genomic alterations in cdRCC and RMC versus ccRCC. In accordance with molecular findings in previous studies, the most common alterations were in SMARCB1 (19% and 67% in cdRCC and RMC respectively), NF2 (14% and 8%), FBXW7 (8% and 8%), CDKN2A (8% and 12%), confirming that they could be therapeutic targets. In this study, the tumor mutational burden (TMB) and microsatellite instability (MSI) biomarkers were also included in the CGP assay; however, the median TMB was low for both subtypes, and none had MSI-high status (1.8 mutations/megabase and 0% for both cdRCC and RMC, respectively).

Recently Gargiuli et al. (27), through integration of multiple datasets, compared cdRCC to UTUC, others RCC and normal tissue. The authors showed that cdRCC was characterized by a distinctive transcriptional program linked to the principal cells of the collecting ducts providing evidence that supports this origin and suggested at least two molecular subtypes of cdRCC distinguished by cell signaling, metabolic and immune-related alterations.

A signature of 31 cdRCC-specific genes was identified, with upregulation in genes related to DNA repair, organelle biosynthesis, innate immune system, interleukin-37, and Rho-GTPases signaling. On the other hand, the downregulated genes were linked to biological oxidations, amino acid metabolism, cell-cell communication, cellular response to external stimuli and G-protein coupled receptors (GPCR). Through the analysis of cancer cell line pharmacogenomics datasets, the authors showed that the gene-signature was predictive of higher sensitivity to some therapeutics options as tyrosine kinase inhibitors, SRC and MAPK signaling inhibitors (27).

To evaluate the intertumoral heterogeneity the authors applied COLA, an approach that enables the identification of consensus clusters combining different gene variability measures and partitioning algorithms. This led the identification of two major consensus clusters: S1 and S2. cdRCC-S1 presented a positive enrichment of gene related to extracellular matrix, innate immunity linked to antimicrobial activity, signaling pathway mediated by GPCR, ion transport and SLC-mediated transport. cdRCC-S2 instead was associated with enrichment of gene related to multiple metabolic pathways, cell cycle, DNA repair, gene expression, programmed cell death, extracellular matrix organization, vesicle mediated transport, adaptive immune-related pathways (T-cell receptor, B-cell receptor, cytokine-signaling, MHC-class I and MHC-class II). No differences for overall immune infiltration and interferon-gene sets were observed (27). Unveiling these processes uniquely enriched or depleted in cdRCC might provide therapeutic options for these patients.

**Figure 2** depicts a summary of the molecular landscape of cdRCC. With the mentioned information, new treatment strategies involving antiangiogenic, metabolic, and immunotherapy were warranted.

## Treatment

Given the rarity of cdRCC, it is difficult to determine the best treatment by robust prospective randomized clinical trials. Reported as the current treatment option for localized disease and also the primary therapy given to cdRCC patients is the surgery. However, the literature related to the type of surgery is limited with insufficient data to recommend any particular technique. There is a consensus that given the aggressive nature of this pathology, the most radical technique possible should be considered (radical nephrectomy plus lymphadenectomy) (28). It constitutes a potential curative treatment as previously reported in case series (29, 30). The role of adjuvant or neoadjuvant therapies is not known.

Sui W et al (10), proved that treatment significantly affected survival. They showed that surgery or surgery plus chemo/radiation conferred a survival benefit over no treatment (HR= 0.13, 95% CI 0.03–0.54 and HR= 0.14, 95% CI 0.03–0.57 respectively); however, chemo/radiation alone was not associated with improved survival compared to no treatment (HR= 0.29, 95% CI:0.05–1.92). The combination of surgery with chemo/radiation did not have additional benefit over surgery alone (HR= 0.74, 95%CI:0.76–1.50) in subgroup analysis.

In the same manner, recently Tang et al. (1) reported a higher benefit in survival in surgery patients than non-surgery patients and between radiotherapy patients and non-radiotherapy patients (a cancer-specific survival of 24 months vs 4 months, p< 0.001; and 8 months vs. 23 months, p< 0.001 respectively). Regarding chemotherapy, patients presenting with stage IV who underwent chemotherapy had higher survival rates than patients without chemotherapy (p= 0.014), but to highlight in this cohort, the combination treatment with surgery plus chemotherapy had higher survival rates than surgery or chemotherapy alone (14 months, 5 months, and 9 months, respectively; p= 0.024). Based on biological similarities between cdRCC and UTUC, previous reports available are based on case reports describing the use of effective regimens and agents in urothelial carcinoma. From the agents described, a limited response was observed to MVAC (methotrexate, vinblastine, doxorubicin, and cisplatin) (31, 32), paclitaxel (33), paclitaxel and carboplatin (34, 35), and doxorubicin and gemcitabine (36).

From a report of nine patients with cdRCC diagnosed by nephrectomy, two patients with metastatic disease showed an objective response and a disease-free survival of 27 and 9 months respectively after first-line treatment with cisplatin and gemcitabine (37). As a result of the above, a prospective phase II study was conducted by Oudard et al. (38) to evaluate the activity of gemcitabine and platinum salts combination in metastatic treatment-naïve cdRCC patients. A total of 23 patients, were treated with gemcitabine and cisplatin or carboplatin for 6 cycles. Objective response rate was 26%, with a median progression-free and overall survival of 7.1 and 10.5 months, respectively. Toxicity was mainly hematological with grade 3–4 neutropenia and thrombocytopenia in 52% and 43% of patients, respectively.

Sheng et al. (39) conducted a prospective, single arm, phase II trial to evaluate the efficacy and safety of sorafenib in combination with gemcitabine and cisplatin followed by maintenance therapy with sorafenib in patients with metastatic cdRCC. A total of 26 patients were treated with this combination. Objective response rate was 30.8%, and the disease control rate was 84.6%. Median progression-free survival and overall survival were 8.8 and 12.5 months, respectively. Relevant toxicities grade 3-4 were mainly leucopenia (26.9%), thrombocytopenia (23.1%), anemia (11.5%) and palmar-plantar erythrodysesthesia (7.7%).

A report of five patients with metastatic cdRCC treated with triple combination of bevacizumab, gemcitabine and platinum salt followed by maintenance therapy with bevacizumab showing an objective response rate of 60% (40). Based on these data, a phase II trial was conducted to evaluate the efficacy and safety of this combination in patients with metastatic medullary RCC and cdRCC (41). From 34 patients enrolled 26 were metastatic cdRCC. Objective response rate was 39%, with a median overall survival of 11 months. However, after an interim analysis, the trial was closed due to toxicity. Grade 3-4 toxicities were reported in 82% of patients, the most common were hematological (20/34) and hypertension (5/34). Two patients had grade 5 toxicity, one with a subdural hematoma and one with encephalopathy.

Several cases have been reported in which patients have achieved partial responses with tyrosin kinasa inhibitors everolimus (42), sunitinib (43, 44), sorafenib (45, 46) and cabozantinib (47).

From a cohort of 384 patients, a small group of 13 metastatic cdRCC patients received first-line treatment with: temsirolimus (2 patients), sorafenib (7 patients), sunitinib (3 patients) and pazopanib (1 patient). For the cdRCC group median overall survival was 4 months, in comparison with 24 months for the non-cdRCC group (48). Only two patients in cdRCC cohort were able to receive a second line of treatment, the sequence were sorafenib-sunitinib and temsirolimus-sunitinib and the overall survival were 49 and 19 months, respectively.

Procopio et al. (49) conducted a prospective, single arm, phase II trial to evaluate the activity and safety of cabozantinib in patients with metastatic cdRCC. The primary endpoint was objective response rate per RECIST. A total of 23 patients were treated with cabozantinib. Median age was 66 years and 83% had undergone a previous nephrectomy. Objective response rate was 35% whereas median progression-free survival and overall survival were 4 months and 7 months respectively. Six patients reported grade 3 adverse events (1 thromboembolic event, 2 arterial hypertension, 2 fatigue and 1 bleeding), while no grade 4-5 adverse events were reported. DNA sequencing was performed, but mature results according to mutational profiles and gene signatures are still awaited.

As previously mentioned, based on the recent data on a transcriptomic profile closer to renal tumors showing high level of immune lymphocyte infiltration (25) and high expression of PD-L1 (50), immunotherapy appears to be an appealing strategy for these patients. There are several case reports of patients treated with nivolumab showing partial response (51–54).

However, prospective data available of immunotherapy trials in cdRCC patients, come from trials with mixed populations that allow the inclusion of these patients. Sternberg et al. (55) conducted a prospective phase III trial to determine the safety and efficacy of atezolizumab in pretreated patients with locally advanced or metastatic urothelial or non-urothelial urinary tract carcinoma. Eight from 1004 patients included, had cdRCC, but unfortunately, data for these patients were not specified. A prospective phase II trial with atezolizumab in combination with bevacizumab in pretreated or treatment-naïve patients included 5 pts with cdRCC, of those 2 (40%) achieved a partial response (56).

Several clinical trials are currently assessing alternative treatment options for patients with variant histology RCC and allow the inclusion of patients with cdRCC (**Table 2**). However, to our knowledge there are no immunotherapy trials specifically for cdRCC.

**Table 2:** Summary of ongoing clinical trials in patients with variant histology renal cell carcinoma (RCC) and allowed collecting duct carcinoma (cdRCC) (57).

Name	ClinicalTrials.gov Identifier	Phase	Intervention arm	Prior Therapy Allowed	Primary Endpoint	Accruing
**CaNI**	**NCT04413123**	II	Cabozantinib plus Nivolumab and Ipilimumab	Yes *	ORR	Yes
**ICONIC**	**NCT03866382**	II	Cabozantinib plus Nivolumab and Ipilimumab	Yes ç	ORR	Yes
**CA209-9KU**	**NCT03635892**	II	Cabozantinib plus Nivolumab	Yes *	ORR	Yes
**ANZUP1602**	**NCT03177239**	II	Nivolumab followed Nivolumab plus Ipilimumab	Yes &	ORR	Yes
**SUNNIFORECAST**	**NCT03075423**	II	Nivolumab plus Ipilimumab versus Standard of Care (sunitinib)	No	OS	Yes
**ALTER-UC-001**	**NCT05124431**	II	Anlotinib plus Everolimus	No	ORR	Yes
** **	**NCT04385654**	II	Toripalimab plus Axitinib	No	PRR	Yes
**RadiCaL**	**NCT04071223**	II	Radium-223 plus Cabozantinib	Yes #	SSE	Yes

ORR, objective response rate; OS, overall survival; PFS, progression-free survival; PRR, pathologic response rate; SSR, symptomatic skeletal event-free survival.

*No prior immunotherapy or cabozantinib.

&No prior immunotherapy.

#No prior cabozantinib.

çNo prior cabozantinib. Also, patients that have received both prior MET or VEGF and prior PD-1/PD-L1/CTLA-4 (sequentially or in combination) are also not allowed.

## Conclusion

The prognostic of cdRCC patients remains poor despite the clinical advances in treatment of metastatic RCC. Surgery is the only potentially curable option in patients with limited disease. Although the treatment with greatest consensus for metastatic disease continues to be the doublet chemotherapy containing platinum salts and gemcitabine, some data are beginning to support the use of other therapeutic agents in this pathology.

The in-dept understanding of the biology of this rare renal cancer subtype has led to knowledge that harbors a characteristic immunogenic and metabolic aberrations, and that targeting these processes might provide therapeutic options for patients.

Given the low incidence, the best strategy to increase knowledge of this pathology requires a multi-institutional effort to design prospective trials evaluating experimental treatments in patients with collecting duct histology.

## Author contributions

All authors listed have made a substantial, direct, and intellectual contribution to the work, and approved it for publication.

## Conflict of interest

JC declares: Employee: Vall d’Hebron University Hospital, Teknon Oncology Department. Consultant and scientific advisory board attendee: Amgen, Astellas, Bayer, BMS, MSD, Johnson & Johnson, Sanofi, Pfizer, Novartis (AAA). Speaker bureau: Asofarma, Astellas, Bayer, Johnson & Johnson, Sanofi. Institutional Studies Collaborations: AB Science, Aragon Pharmaceuticals, Arog Pharmaceuticals, INC, Astellas Pharma, Astrazeneca AB, Aveo Pharmaceuticals INC, Bayer AG, Blueprint Medicines Corporation, BN Immunotherapeutics INC, Boehringer Ingelheim España, S.A., Bristol-Myers Squibb International Corporation (BMS), Clovis Oncology, INC, Cougar Biotechnology INC, Deciphera Pharmaceuticals LLC, Exelixis INC, F. Hoffmann-La RocheLTD, Genentech INC, Glaxosmithkline, SA, Incyte Corporation, Janssen-Cilag International NV, Karyopharm Therapeutics INC., Laboratoires Leurquin Mediolanum SAS, Lilly, S.A., Medimmune, Millennium Pharmaceuticals, INC., Nanobiotix SA, Novartis Farmacéutica, S.A., Pfizer, S.L.U, Puma Biotechnology, INC, Sanofi-Aventis, S.A., SFJ Pharma LTD. II, TevaPharma S.L.U. CS declares: Advisory or Consultancy rol: Astellas Pharma, Bristol Myers Sqquib, EUSA Pharma, Ipsen, Novartis, Pfizer, Sanofi, Merck Sharp & Dohme Corp. Speaker Bureau/Expert Testimony: Astellas Pharma, Bristol Myers Sqquib, Ipsen, Pfizer, Astrazeneca. Institutional financial interests: AB Science, Aragon Pharmaceuticals, Arog Pharmaceuticals, INC, Astellas Pharma., Astrazeneca AB, Aveo Pharmaceuticals INC, Bayer AG, Blueprint Medicines Corporation, BN Immunotherapeutics INC, Boehringer Ingelheim España, S.A., Bristol-Myers Squibb International Corporation (BMS), Clovis Oncology, INC, Cougar Biotechnology INC, Deciphera Pharmaceuticals LLC, Exelixis INC, F. Hoffmann-La Roche LTD, Genentech INC, Glaxosmithkline, SA, Incyte Corporation, Janssen-Cilag International NV, Karyopharm Therapeutics INC., Laboratoires Leurquin Mediolanum SAS, Lilly, S.A., Medimmune, Millennium Pharmaceuticals, INC., Nanobiotix SA, Novartis Farmacéutica, S.A., Pfizer, S.L.U, Puma Biotechnology, INC, Sanofi-Aventis, S.A., SFJ Pharma LTD. II, Teva Pharma S.L.U. Other: Research Grant/Funding (Self): Ipsen. Travel expenses: Hoffmann-La Roche LTD, Pfizer, Ipsen

RMB: (all unrelated in the last 3 years): Consulting or advisory and/or speakers bureaus for Sanofi Aventis, AstraZeneca, Merck Sharp & Dohme, Astellas, BMS and received travel and accommodations expenses from Roche, Sanofi Aventis, Astellas, Janssen, Merck Sharp & Dohme, Bayer, and Pfizer.

The remaining authors declare that the research was conducted in the absence of any commercial or financial relationships that could be construed as a potential conflict of interest.

## Publisher’s note

All claims expressed in this article are solely those of the authors and do not necessarily represent those of their affiliated organizations, or those of the publisher, the editors and the reviewers. Any product that may be evaluated in this article, or claim that may be made by its manufacturer, is not guaranteed or endorsed by the publisher.
